# Optimization of patient radiation protection in pelvic X‐ray examination in Ghana

**DOI:** 10.1120/jacmp.v13i4.3719

**Published:** 2012-07-05

**Authors:** Eric K. Ofori, William K. Antwi, Diane N. Scutt, Matt Ward

**Affiliations:** ^1^ Department of Radiography School of Allied Health Sciences, College of Health Sciences, University of Ghana Korle‐Bu‐Accra Ghana; ^2^ School of Health Sciences University of Liverpool United Kingdom; ^3^ Integrated Radiological Services Limited Liverpool United Kingdom

**Keywords:** patient radiation protection, gonadal dose, patient dose audit

## Abstract

Pelvis X‐ray examinations inevitably involve exposure of the gonads to ionizing radiation. In line with the principle of keeping doses as low as reasonably practicable (ALARP), accurate patient dose measurement is vital if we are to ascertain that these exposures are fully optimized. The study aimed to provide patient dose estimates for pelvis examination being undertaken at 10 separate hospitals in Ghana in order to provide an initial quantitative indication of each site's typically achievable radiation safety and quality standards. The method employed was adapted from established methods and peer reviewed literature, such as the International Atomic Energy Agency (IAEA) publications on optimization of the radiological protection of patients undergoing radiography, fluoroscopy, and computed tomography examinations in some countries in Africa, Asia, and Eastern Europe. Dose measurements were calculated on 323 patients (137 (42%) male, 186 (58%) female, ages, 38.56 yr ±9.0; range 20–68). The entrance surface dose (ESD) was determined by an indirect method, using the patient's anatomical data and exposure parameters utilized for the specific examination. The Quality Assurance Dose Database software (QADDs) developed by Integrated Radiological Services Ltd. in Liverpool, UK was used to generate the ESD values. The study identified variations in the technique factors used compared with the recommendations in the European Commission (EC) quality criteria. Eighty percent of the hospitals recorded lower ESD values below IAEA recommended diagnostic reference levels (10 mGy) and 40% of the hospitals exceeded the UK national reference value (4 mGy). However, one hospital consistently recorded higher ESDs than the other hospitals. The variations in the data recorded demonstrate the importance of creating awareness by the radiographic staff on quality assurance and standardization of protocols to ensure satisfactory standards and optimized radiation dose to patients and staff.

PACS number: 87.59.B

## I. INTRODUCTION

Protecting the gonads of children and adults is of particular importance during diagnostic imaging of the pelvis. Evidence suggests that X‐rays could cause direct damage to the gonad which could result in mutation.^(^ ^1^ ^)^ Gonad shielding during diagnostic X‐ray procedures is an effective way of reducing dose to patients' reproductive organs and reduces the risk of genetic effects in future generations.^(^ ^2^ ^)^ Given the unavoidable harmful potential effects associated with exposure to ionizing radiation, it is important not just to provide gonad shielding, but also to measure patient doses, and reduce them where possible.

Over the years, reductions in patient doses have been achieved through advances in technology and changes in clinical practice. In the United Kingdom in particular, repeated national dose surveys by the UK Health Protection Agency (HPA) (formerly, the National Radiological Protection Board) have shown a significant lowering of patient dose for individual procedure types.^(^ ^3^ ^,^ ^4^ ^)^ However, whilst some dose reduction measures have a positive effect on image quality, others degrade contrast or increase noise. Thus, it is important not just to reduce doses but to optimize each imaging technique, maximize its efficiency, and determine the right balance between patient dose and image quality. Once an X‐ray examination is definitely justified, the principle of optimization implies that during the examination, the margin of good over harm is maximized by giving attention to all aspects of radiographic examination process (i.e., there is net benefit to the patient).^(^ ^5^ ^)^

The most reliable dosimetry quantities commonly used in diagnostic radiology to give an indication of the typical dose that is being delivered to an average adult patient are the patient entrance surface (skin) dose (ESD) including backscatter for simple X‐ray projections, and the dose area product (DAP) for complex examinations.^(^ ^6^ ^,^ ^7^ ^)^ The ESD, in particular, is recommended as the most appropriate dosimetry quantity for simple X‐ray projections since it meets the three basic conditions set out by the International Atomic Energy Agency (simple to measure, permits direct measurement on patient during the examination, and is representative of the dose received by the patient). It is also recommended by the Commission of the European Communities (CEC) in the document on quality criteria for the most common radiographic images. In addition, the measurement of ESD permits easy comparison with published diagnostic guidance or reference levels.^(^ ^7^ ^,^ ^8^ ^,^ ^9^ ^,^ ^10^ ^)^

In Ghana, patient radiation protection in pelvis X‐ray examination has not been given much attention. The aim of this study, therefore, was to estimate patient dose in pelvis examination from 10 imaging rooms as an indicator of the status of patient radiation protection. The estimated mean ESD values were compared with the International Atomic Energy Agency,^(^ ^9^ ^)^ the European Commission (EC) guidance on diagnostic reference levels for medical exposures,^(^ ^11^ ^)^ and the 2005 United Kingdom reviewed reference levels.^(^ ^4^ ^)^ This comparison was felt to be appropriate because at the time of the study, there were no accepted local or national diagnostic level values in Ghana for comparison. The ESD was determined from the radiographic examinations of adequate image quality of patients who underwent the pelvis examinations during the study. ESDs were calculated from the patient's anatomical data and exposure parameters utilized for the specific examination.

## II. MATERIALS AND METHODS

The basic methodology adopted and adapted for the study was that used in the Coordinated Research Project (CRP) by the International Atomic Energy Agency (IAEA) on optimization of the radiological protection of patients undergoing radiography, fluoroscopy, and computed tomography examinations in some countries in Africa, Asia and Eastern Europe.^(^ ^9^ ^)^ The IAEA study measured the entrance surface dose (ESD) using thermoluminescent dosimeters (TLDs) placed on the patient's skin at the center of the X‐ray beam. In this current study, the ESD was determined for each patient undergoing radiographic pelvis examinations that were judged locally at each site by individual operators to have achieved adequate image quality for diagnosis. The ESD was determined from the generator output after air kerma readings were taken using a calibrated solid state detector Unfors Xi Platinum (SN: 127871; Unfors Inc., Billdal, Sweden) placed at one‐meter focus detector distance (FFD) on top of the table at different kVp settings. This option of dosimetry was chosen because of the difficulties involved in obtaining and using TLD dosimeters in Ghana. It was also easy to access the solid state detector, which was also used for other quality control tests.

### A.1 Subjects

Radiation dose assessment was conducted on 323 patients during the study period. Inclusion criteria were patients over 18 years who underwent pelvis examinations in the selected hospitals. The pelvis examination was selected for this study because during this examination, critical organs (testes, ovaries) that contribute to effective dose are irradiated. Data was collected on 323 patients who underwent antero–posterior (AP) pelvis examination in the 10 selected hospitals. Ten radiographers and ten radiographic technicians participated in the study and completed the data collection sheets after each examination. The examination rooms were chosen for practical and logistical reasons, and were representative of the regional and district hospitals in Ghana. The selected imaging rooms are shown in Table 1.

**Table 1:** Imaging rooms involved in the study.

*Code*	*Hospital Names*
KATH3	Komfo Anokye Teaching Hospital Room 3
KATH2	Komfo Anokye Teaching Hospital Room 2
KBPOLY	Korle‐Bu Teaching Hospital Polyclinic
RIDGE	Ridge Regional Hospital
KBMR3	Korle‐Bu Teaching Hospital Room 3
KBACC	Korle‐Bu Accident Centre
SUNT	Suntreso Government Hospital
KNUST	KNUST Hospital
37MILT	37 Military Hospital
TRUST	Trust Hospital

### A.2 X‐ray equipment and intensifying screen–film combination

Table 2 shows the characteristics of the X‐ray machines in the ten rooms used for the study. All of these were constant potential generators with 2.5 mm Al total equivalent filtration at 80 kVp. Two manufacturers' cassettes (Agfa and Kodak) were used with screen–film combination speed of 400. Since the study was aimed to provide patient dose estimates based on the patient's anatomical data and exposure parameters utilized for the specific examination, the performance of these cassettes was not assessed. The ESD was determined from the radiographic examinations, which were of adequate image quality.

**Table 2:** Features of X‐ray machines in the ten examination rooms used for the study.

		*Characteristics of the X‐ray Generator*		
*X‐ray Room*	*Type*	*Manufacture Date*	*Power Rating (kVp)*	*Exposure Setting*
KATHR3	Siemens	2002	30–150	AEC and Manual
KATHR2	Siemens	2002	30–150	AEC and Manual
KBPOLY	Philips	1997	40–150	AEC and Manual
RIDGE‐R1	Philips	1998	40–150	AEC and Manual
KBMR3	Shimadzu	1992	40–125	Manual
KBACC	Siemens	2002	30–150	AEC and Manual
SUNT‐R1	Philips	1997	40–150	AEC and Manual
KNUST‐R1	Philips	1996	40–150	AEC and Manual
TRUST‐R1	Philips	2005	40–150	AEC and Manual
37 MILT	Siemens	1999	30–150	AEC and Manual

### B.1 Patient physical measurements

The anatomical thickness (cm), weight (kg), height (m), and gender of the patients, as well as radiographic exposure factors (kV and mAs) used for each patient's examination, were recorded. Patients who met the inclusion criteria (aged 18 years and above) in the selected hospitals were weighed and a specially designed caliper of least count of 1 mm was used to measure the anatomical thickness for the body part under examination, which in turn was used to estimate the focus skin distances for the examination. The anatomical thicknesses of the patient were measured at the level of anterior–superior iliac spines. A tape measure of least count 0.1 cm was used to measure the focus film distances (FFD). All FFD measurements were from the center of the tube to the film or the table top. The datasheets were placed near the console of the X‐ray room and were completed by the radiographic staff for each patient requiring pelvis examination.

### B.2 Quality control tests

Quality control (QC) tests were performed concurrently on the X‐ray equipment located in the selected rooms, in accordance with the standard procedures/protocols in the American Association of Physicists in Medicine (AAPM) Report 74^(^ ^12^ ^)^ and the Institute of Physics and Engineering in Medicine (IPEM) Report 91,^(^ ^13^ ^)^ and the results are presented in Table 3. Tube output and filtration were assessed with a calibrated solid state detector Unfors Xi Platinum and high‐purity aluminum HVL attenuator set (model GAMMEX RMI 115A, Gammex Inc., Middleton, WI). The accuracy, reproducibility of kVp (i.e., kVp calibration), and timer were also measured using the detector. For the light‐radiation beam alignment test, two test tools were used; the RMI Collimator Test Tool (model 161B, Computerized Information Technology Ltd. (CIT), Milton Keynes, UK) designed to evaluate the collimator according to National Centre for Devices and Radiological Health (NCDRH) specifications and the Beam Alignment Test Tool (model 162A, CIT, UK), which was used with the Collimator Test Tool to provide a simple test for beam alignment. The spectral emission of the intensifying screen was determined by exposing an open cassette under subdued light condition and the emission from the screen was then observed. For many years, calcium tungstate screens (${\text{CaWO}}_{4}$) were the most common intensifying screens used in Ghana but, over the last ten years, most diagnostic imaging departments have switched to the use of rare earth screens. These have features of greater dose reduction and improved image quality as a result of their more efficient absorption of X‐ray quanta. Presently, most films imported into the country are green‐sensitive and initially cassettes with green emitting screens were supplied. It was observed during field trips that most imaging departments have both green‐ and blue‐emitting screens. Since these cassettes were old and often were supplied before the radiographer in charge was on staff, they are often used interchangeably, causing problems with resultant poor image quality and subsequently repeated films and increased patient dose.

**Table 3:** Summary results of QC test on X‐ray equipments in all rooms.

		*X‐ray Tube Rooms*									
		*Percentage Deviation (%)*									
*Reference*	*Parameters and Tolerance Levels*	*KATH‐R3*	*KATH‐R2*	*KBPOLY*	*RIDGE*	*KBMR3*	*KBACC*	*SUNT*	*KNUST*	*37MILT*	*TRUST*
IPEM 91	kV accuracy	2.10	2.40	2.30	1.50	2.30	3.10	4.20	3.10	4.20	0.70
RAD12	(10%)										
TGR32	HVL, mm Al (at 80 kVp)	3.22	2.81	3.39	3.13	3.39	3.12	3.38	3.22	3.52	3.16
TGR32	Total filtration	3.12	2.42	3.40	2.93	3.40	2.93	3.40	3.12	3.70	3.03
IPEM RADII	Timer accuracy (10%) (100–1000 ms)	N/A (mAs)	9.00	0.30	0.30	0.30	N/A (mAs)	0.10	0.30	1.80	0.30
TGR32	Timer reproducibility (5%) (100–1000 ms)	N/A (mAs)	9.00	0.30	0.30	0.30	N/A (mAs)	0.10	0.30	1.80	0.30
TGR32	Output linearity (mGy/mAs) (5%)	1.80	1.80	2.00	1.90	2.00	1.80	2.00	1.90	1.90	1.90
IPEM 91 RAD09	Radiation output repeatability (10%)	0.30	0.10	0.70	3.10	0.70	0.60	0.10	0.10	0.30	0.05
IPEM 91 RAD09 Output reproducibility (rem 20%)	Radiation	0.76	6.55	1.13	0.51	1.13	1.50	1.03	0.52	4.98	1.06

### B.3 Patient radiation dose assessment

The entrance surface dose (ESD) for patients was assessed by indirect method, using data of radiation output of the X‐ray tubes and exposure factors (kVp and mAs). In order to determine the ESD from the generator output, air kerma values at different kVp settings were first measured using a calibrated solid state detector, Unfors Xi Platinum. The detector was placed at one‐meter focus detector distance on top of the table at different kVp settings. The focus patient surface distance (FSD) and radiographic exposure factors (kVp and mAs) used for selected examinations were recorded on a self‐designed Excel sheet. Datasheets were collected on a weekly basis, and the exposure factors recorded were cross‐checked against actual practice with the radiographers who recorded them, in order to validate the figures. The data were entered into Excel and transferred into the Quality Assurance Dose Database software (QADDS) for computation of the ESD. The accuracy of a given TLD measurement (i.e., the difference between the indicated and true dose values) depends on the nonrandom or systematic uncertainties in the method for converting the TLD response (count) to dose. The UK Health Protection Agency (HPA),^(^ ^14^ ^)^ suggests that when account is taken of all influence quantities and all available correction factors used, the overall uncertainty of the readings should be ≤±25% at the 95% confidence level. Studies^(^ ^15^ ^,^ ^16^ ^)^ have shown that ESDs calculated using QADDs are within ±20% of ESDs measured using thermoluminescent dosimeters (TLDs).

In order to perform calculations of ESD, several items of information such as kV selected, mAs delivered, and the focus‐to‐skin distance (FSD) were entered into the front page datasheet of the program. The program performed the calculation of ESD using the formula:

(1) $$\text{ESD}=\text{BSF}\times \text{Tube}\;\text{Output}\;(\mu \text{Gy}/\text{mAs})\times \left(\frac{100}{\mathrm{FSD}}\right)\;{\;}^{2}\times \text{mAs}$$

where BSF is the backscatter factor = (1+ backscatter fraction), and FSD is the focus‐to‐skin distance used.

Dose measurements were calculated on 323 patients of which 137 (42.4%) were males and 186 (57.6%) were females. An Excel output data file was then generated from the QADDS which were converted into an SPPS version 16 file to facilitate descriptive and inferential analysis.

## III. RESULTS

### A. Descriptive statistics of examinations and patient data

Descriptive statistics on patients' age, weight, body mass index, and the body part thickness are shown in Table 4. The age range for all patients in the hospitals was 20.0–68.0 years, with mean and standard deviation values as 38.6 yrs and 9.0, respectively.

**Table 4:** Descriptive statistics of the participants.

		*Gender*		
*Demographic Data*	*Descriptive Statistics*	*Male (* N=137 *)*	*Female (* N=186 *)*	*Average Values for All Patients*
Age (yrs)	Min	25.0	20.0	20
	Max	68.0	68.0	68
	Mean±SD	38.7±8.9	38.5±9.1	38.56±9.0
Weight (Kg)	Min	58.0	55.0	55
	Max	113.0	116.0	116
	Mean±SD	84.6±11.5	77.9±13.3	80.7±13.0
Body Mass Index (${\text{kgm}}^{-2}$)	Min	20.0	19.3	19.3
	Max	39.8	44.2	44.20
	Mean±SD	29.1±3.9	30.6±4.8	30.0±4.5
Patient Thickness (m)	Min	0.2	0.2	0.2
	Max	0.3	0.4	0.4
	Mean±SD	0.3±0.02	0.39±0.03	0.33±0.02

The EC criteria for ESD calculations assume a 20.0 cm AP trunk thickness and average weight of 70.0 kg for a standard adult patient. However, in this sample of Ghanaian patients, the range of AP trunk thicknesses for pelvis was 20.0−40.0 cm. This influenced focus‐to‐skin distance used to calculate ESD because, if the focus‐to‐film distance (FFD) is constant, then a range of patient size will present a corresponding range of FSD values, which have a direct impact on ESD.

### B. Analysis of entrance surface dose (ESD)

The mean and the standard deviation (SD) of the entrance surface doses (ESD) estimated for the individual examinations for all the ten rooms in addition to the range factor (RF) — defined as the ratio of maximum to minimum dose for the same type of examination — were calculated and are presented in Table 5.

**Table 5:** Mean and standard deviation (SD) values for ESD, minimum, maximum, and range factor of ESD within rooms and range factor of ESD values for the same type of examination between the rooms for pelvis examinations across hospitals compared with UK‐2005 and EU/IAEA‐recommended values.

	*ESD (mGy) for Individual Examinations in All Rooms*				*Range Factor Between Rooms*		*DRL for UK and EU/IAEC*	
*Hospitals*	*Mean (SD)*	*Min*	*Max*	*RF*	*Based on mean ESD values*	*Based on Max/Min ESD values*	*UK‐2005*	*EU/IAEA*
KATH‐R3	2.3 (0.6)	1.4	3.6	2.6				
KATH‐R2	2.7 (1.2)	1.3	4.5	3.5				
KBPOLY	2.3 (1.4)	0.6	4.7	7.8				
RIDGE	4.9 (1.9)	3.1	10.1	3.3				
KBMR3	2.9 (0.9)	1.2	4.4	3.7	14.8	69.8	4	10
KBACC	3.3 (0.7)	0.9	4.4	4.9	
SUNT	32.5 (4.9)	19.8	41.9	2.1				
KNUST	4.7 (2.2)	2.3	9.1	4.0				
37 MILT	2.2 (1.0)	1.1	4.6	4.2				
TRUST	10.7 (6.4)	5.0	24.5	4.9				

The range factor, which highlights the spread/variation in the ESD values for the same type of examination either within or between the rooms, as well as the minimum, maximum, and range factor of ESD values for the same type of examination in the same room (intraroom variation) are also shown in Table 5. In this way, the factor by which the dose of radiation can vary for the same examination in the same room is indicated by the quotient of highest and lowest dose for an examination.

### C. Summary of radiographic exposure data

The mean and the range of the exposure factors (the tube voltage (kVp), the tube current (mAs), and the focus film distance (FFD) in centimeters for the individual hospitals are presented in Table 6, alongside the European Commission's recommended exposure factor values^(^ ^11^ ^)^ for good radiographic techniques.

**Table 6:** Recommended range values for good radiographic techniques in EUR16260 compared with mean (range) exposure factor values for pelvis AP examination.

	*Radiographic Data for Ghana*				*EUR 16260 Recommended Radiographic Data For:*			
*Hospital*	*Tube Voltage (kVp)*	*Tube Current (mAs)*	*FFD (cm)*	*Screen Film System (nominal speed class)*	*Tube Voltage (kVp)*	*Tube Current (ms)*	*FFD (cm)*	*Screen Film System (nominal speed class)*
KATH‐R3	72.29(70.0–75.0)	19.11(16.0–25.0)	100					
KATH‐R2	79.80(75.0–83.0)	15.4(10.0–22.0)	100					
KBPOLY	59.02(52.0–66.0)	33.42(13.6–58.0)	100					
RIDGE	75.3(68.0–90.0)	37.2(20.0–50.0)	100					
KBMR3	67.25(56.0–73.0)	33.2(20.0–40.0)	100	400	75–90	<400	115 (100–150)	) 400
KBACC	75.2(60.0– 83.0)	22.7(10.0–25.0)	100	
SUNT	58.56(50.0–63.0)	578.08(578.0–579.0)	100					
KNUST	82.93(75.0–90.0)	31.9(22.0–50.0)	100					
37 MILT	75.8(65.0–83.0)	17.38(10.0–32.0)	95					
TRUST	56.57(50.0–63.0)	149.67(80.0–250.0)	100					

Note: All the rooms in the study employed two‐knob technique settings where only kVp and mAs were selected as against the case of three‐knob techniques settings (kVp, mA and s).

### D. Summary of the analysis of quality control tests on radiographic equipment

The summary results of quality control (QC) checks done on X‐ray equipment are presented in Table 3. By comparing these results with the recommended tolerances in IPEM Report 91,^(^ ^13^ ^)^ it can be seen that all the X‐ray machines used in the study passed all QC tests performed.

## IV. DISCUSSION

A total of 323 dose measurements on antero–posterior pelvis examinations were recorded during the study. The proportion of males (46.7%) to females (53.3%) in this study reflects the demography in Ghana where females constitute about 55% of the population.^(^ ^17^ ^)^ In the EC quality criteria and the IPEM Report 91,^(^ ^13^ ^)^ it is recommended that the ESD measurements be made on statistically significant sample of patients (minimum 10) whose weights are near the standard adult patient of average weight 70.0 kg ±10. This study complied with this recommendation and therefore the estimate of ESDs for the various examinations could be considered sufficiently representative value for the specific rooms.

This study has provided some initial base line data on the size of the average adult patient in Ghana and the corresponding dose for pelvis examinations. The mean weight recorded for all patients was 80.72±13.0kg. This is different from the mean weight recorded in the IAEA study in 2004^(^ ^9^ ^)^ on patients undergoing radiographic examinations in some European and Asian countries. In the IAEA study, an average weight of 70.0 kg ±10 was considered appropriate for the European participating countries, while 65±10kg was used for the Asian countries. The average weight of the only African country that participated in the study, Morocco, was not stated, but a compromise was made to enable a comparison of the measured doses with reference doses. It is therefore relevant to compare the estimated dose recorded from this study with the reference values based on the average weight in the Ghanaian context. The fact that the weight of the Ghanaian average man differs considerably from the European and Asian male suggests that more applicable data are needed for the Ghanaian situation.

The radiographic technique parameters recorded show that there were variations in the technique factors when compared with the recommendations in the EC quality criteria.^(^ ^11^ ^)^ Varying radiographic voltages and reduced focus film distances were used. All these factors have adverse influence on the outcome of the dose to patient. The above problem was not isolated to Ghana, but is common in other developing countries.^(^ ^18^ ^,^ ^19^ ^,^ ^20^ ^)^ These problems probably could partly be associated with the inadequate training of imaging staff, variation in patient physique, different types of equipment, and variety of techniques used in different hospitals. Also, the different methods of documentation of data on radiation dose could also lead to apparent dose variations.^(^ ^20^ ^,^ ^21^ ^)^

This study also revealed that there were inconsistencies in the use of the focus film distances as recommended in the EC quality criteria. The EC criteria recommend an average FFD of 115 cm and a range of 100–150 cm. Most hospitals used FFD values below the average values (115 cm) but equal to the minimum recommended value (100 cm). Since ESD is inversely proportional to the square of the FFD, for the same kV and mAs the dose reaching the patient is expected to be high. Although the general trend across all centers is the use of lower FFDs and this, in part, might explain higher ESDs, it can be seen that the results do not show this as a universal trend (some centers with low FFDs present mean ESDs around 2 mGy, some much, much higher). It is worth noting that changing FFD could be a good change, but will still not solve all discrepancies found in the study. It is therefore essential that policies on quality control and assurance monitoring programs be enforced in the hospitals to protect the patient against unnecessary exposures through repeat examinations.

Generally, ESD values for the same type of examination in the same room will vary due to the differences in patient size and in the radiographic technique used by different radiographers. Variations in the ESD values between different X‐ray rooms will additionally be due to differences in radiographic equipment, film type, processing, chemistry, and processing conditions. The mean ESD values for the individual examinations varied considerably across all hospitals and within hospitals. A particular hospital, Suntreso, recorded consistently higher ESDs than the other departments. On closer investigation, it was revealed that the automatic exposure control (AEC) device was consistently being incorrectly used or was frequently overridden by the radiographer for no apparent reason. Automatic exposure devices are intended to take some of the human error out of exposure factor selection, but overriding them has a detrimental effect on patient dose. This particular issue (of not using AEC where they were available) was not confined to this hospital; some hospitals with AECs had disconnected them but the reasons for this were not clear and warrant further investigation. It is likely that staff had not been trained in the use of automatic exposure devices, lacked confidence, and therefore defaulted to the original methods of manual selection of exposure factors. The lower doses recorded in the other hospitals in comparison with Suntreso was because lower mAs were used.

The above results suggest that hospitals with lower ESD values than the reference dose values provided by UK, EC, and IAEA are acceptable in terms of dose to the patient. However, optimization has to be interpreted in the light of the number of the acceptable radiographs produced with adequate image quality. The use of lower ESD values in pelvic examinations is encouraging but should not be at the expense of image quality. There should be always a balance between the patient dose and the quality of the radiographic images produced.

There was a considerable variation in the range factor for ESD for the same type of examination in the same room. The range factor highlights the spread/variation in the ESD values for the same type of examination either within or between the rooms. This implies that in the same X‐ray room there were variations in radiographic technique between radiographers (usually differences in exposure time) which could be, in part, related to differences in patient size, but could not be accounted for by this parameter alone. In terms of interroom variations, the mean ESDs showed variations in dose between rooms. The ratio of the maximum dose in one room to the minimum dose in another was 69.8. These variations in the ESD for the same type of examination between the rooms may be due in part to the different technical characteristics of radiographic equipment, but are mainly due to the techniques employed, and particularly technique inconsistency. These inter‐ and intrahospitals' dose variations for the same type of examination confirm the variability in the operational conditions within and among the hospitals.

The results of quality control (QC) checks done on X‐ray equipment compared with the recommended tolerances revealed that all the X‐ray machines used in the study passed the entire range of QC test undertaken. However, in clinical situations where diagnostic conditions for which equipment‐, human‐, and technique‐related factors are variable, the use of these X‐ray machines may still lead to unoptimized diagnostic procedures. Therefore the QC results provide guidance on the choice of exposure techniques and suggest the need for optimized dose reduction methods. The lowering of mAs has been found to be effective for X‐ray equipment with better timer reproducibility than lower timer reproducibility. Increased filtration has also been found to be effective for an X‐ray machine of lower half value layer. Likewise, the increasing of tube potential reduced the patient dose more efficiently for X‐ray machines that possessed better tube voltage consistency and higher accuracy.

Other related factors, such as exposure charts and documented protocols, were lacking in all the rooms in the study. This obviously contributes to inappropriate and inconsistent kVp settings employed in the hospitals relative to the recommended values in the EC quality criteria used in the hospitals. The absence of all of these have the obvious consequence of unnecessary exposure to the patient. There was a general lack of awareness of the importance and significance of radiation protection issues prevalent at all stages of the study, and this is a direct consequence of inadequate training on such matters at all levels.

Ghana is not alone in experiencing these problems: similar findings were also identified in Nigerian hospitals, leading to higher radiation doses in some hospitals. A study in Nigeria in sub‐Saharan Africa^(^ ^22^ ^)^ which assessed the accuracy in patient positioning, beam collimation, and identification of radiographs as indicators for quality assurance and radiation protection, showed that positioning and beam collimation contributed to a high percentage of unnecessary radiation dose to the patients in all the participating hospitals. The authors suggested that poor practice of radiation protection exists in the hospitals in Nigeria, and that most patients were at risk of having other parts of the body not involved in the required X‐ray examination exposed to radiation. A similar study, also in Nigeria,^(^ ^23^ ^)^ revealed that inappropriate technique and exposure factors contribute 33.9% to film wastage in some Nigerian hospitals. A study conducted in Ghana in 1998,^(^ ^24^ ^)^ which assessed the radiation doses to patients in selected X‐ray examinations in Ghana, attributed the cause of high patient dose to the absence of formal QA and QC programs to ensure optimum performance of the X‐ray facilities. Currently, there are no national agreed dose levels consistent with the international dose averages in Ghana. Moreover, there is no evidence to suggest that the Economic Communities of West African States, of which Ghana is a member, has any recommended dose levels which member states should follow.

## V. CONCLUSIONS

The study showed variations in technique, exposure factors, film–screen combinations, and radiation dose for the same type of examination, both within and between rooms, which strongly supports the idea that further optimization is possible. Eighty percent of the hospitals recorded lower ESD values below EC/IAEA recommended diagnostic reference levels (10 mGy), and 40% of the hospitals exceeded the UK national reference value (4 mGy). Radiographic practices in Ghana are not fully optimized and this, therefore, calls for robust implementation of an appropriate and realistic QA program, which currently is not in existence in all the facilities surveyed. The variations in the data obtained also demonstrate the importance of creating awareness for the radiographic staff about regular quality control testing of the equipment and standardization of protocols, the urgent need for intervention and appropriate corrective actions in order to improve and standardize practice, enhancing the quality of the radiographs, and avoiding unnecessary risks of increased radiation dose to patients and staff. Also these variations, which are assumed to be present in most of the X‐ray departments operating in the country, point to the need for the introduction of a national protocol and QA system, and frequent dose audits. A continuing need for bringing radiological procedures in Ghana in line with the “European Guidelines on Quality Criteria for Diagnostic Radiological Images”^(^ ^11^ ^)^ is worth emphasizing, in order to protect patients and staff from unnecessary radiation dose.

## ACKNOWLEDGMENTS

The authors acknowledge the support and cooperation received from the staff of the participating hospitals, and are grateful to Professors Edwin Wiredu and Cyril Schandorf for their technical support.
